# Genome-wide characterization and evolution analysis of miniature inverted-repeat transposable elements in Barley (*Hordeum vulgare*)

**DOI:** 10.3389/fpls.2024.1474846

**Published:** 2024-10-31

**Authors:** Ruiying Li, Ju Yao, Shaoshuai Cai, Yi Fu, Chongde Lai, Xiangdong Zhu, Licao Cui, Yihan Li

**Affiliations:** ^1^ College of Bioscience and Engineering, Jiangxi Agricultural University, Nanchang, Jiangxi, China; ^2^ The Public Instrument Platform of Jiangxi Agricultural University, Jiangxi Agricultural University, Nanchang, China

**Keywords:** barley, MITEs, miRNA, amplification, domestication

## Abstract

Miniature inverted-repeat transposable elements (MITEs) constitute a class of class II transposable elements (TEs) that are abundant in plant genomes, playing a crucial role in their evolution and diversity. Barley (*Hordeum vulgare*), the fourth-most important cereal crop globally, is widely used for brewing, animal feed, and human consumption. However, despite their significance, the mechanisms underlying the insertion or amplification of MITEs and their contributions to barley genome evolution and diversity remain poorly understood. Through our comprehensive analysis, we identified 32,258 full-length MITEs belonging to 2,992 distinct families, accounting for approximately 0.17% of the barley genome. These MITE families can be grouped into four well-known superfamilies (*Tc1/Mariner-like*, *PIF/Harbinger-like*, *hAT-like*, and *Mutator-like*) and one unidentified superfamily. Notably, we observed two major expansion events in the barley MITE population, occurring approximately 12-13 million years ago (Mya) and 2-3 Mya. Our investigation revealed a strong preference of MITEs for gene-related regions, particularly in promoters, suggesting their potential involvement in regulating host gene expression. Additionally, we discovered that 7.73% miRNAs are derived from MITEs, thereby influencing the origin of certain miRNAs and potentially exerting a significant impact on post-transcriptional gene expression control. Evolutionary analysis demonstrated that MITEs exhibit lower conservation compared to genes, consistent with their dynamic mobility. We also identified a series of MITE insertions or deletions associated with domestication, highlighting these regions as promising targets for crop improvement strategies. These findings significantly advance our understanding of the fundamental characteristics and evolutionary patterns of MITEs in the barley genome. Moreover, they contribute to our knowledge of gene regulatory networks and provide valuable insights for crop improvement endeavors.

## 1 Introduction

Transposable elements (TEs) are mobile DNA sequences that can move within and between eukaryotic genomes, where they often constitute a large and dominant fraction. For instance, maize (*Zea mays*) and common wheat (*Triticum aestivum*) genomes are composed of 80% and 85% TEs, respectively (Perumal et al., 2020; Li Y. et al., 2022). By inserting into new genomic locations, TEs can induce genome rearrangements and affect chromosome structure, genome size, and gene expression (Flutre et al., 2011). TEs are classified into two primary classes according to their transposition mechanisms: Class I TEs (retrotransposons) and Class II TEs (DNA transposons) (Dhillon et al., 2014). Class I TEs transpose via an RNA intermediate and a ‘copy-and-paste’ mode, while Class II TEs transpose via a DNA intermediate and a ‘cut-and-paste’ mode (Loot et al., 2006).

Miniature inverted-repeat transposable elements (MITEs) are non-autonomous Class II TEs that rely on the transposase enzymes encoded by their autonomous counterparts (Klai et al., 2022). MITEs are characterized by: short lengths of 50 to 800 base pairs (bp); the presence of terminal inverted repeats (TIRs ≥ 10 bp) and target site duplications (TSDs, 2–10 bp) at both ends; a high A/T abundance, which facilitates the formation of secondary structures; the absence of an open reading frame and the inability to encode transposase enzymes; and some MITEs can also transcribe double-stranded RNAs that can be processed into small RNAs (sRNAs) with regulatory functions (Wang et al., 2009; Han et al., 2010; Lu et al., 2012; Guo et al., 2017). The first MITEs were identified in the *Z. mays* mutant allele *WAXY* (*Wx*-*B2*), which contains a 128 bp insertion with 14 bp TIRs (5’-GGCCTTGTTCGGTT-3’) and 3 bp TSDs (TAA/TTA) at the 5’ and 3’ ends, respectively (Pegler et al., 2023). Most MITEs originate from autonomous Class II TEs, such as *Tc1/Mariner-like*, *PIF/Harbinger-like*, *hAT-like*, *Mutator-like*, and *CACTA-like* elements, based on the similarity of their TIRs and TSDs (Han et al., 2013; Guo et al., 2022). In plants, the *Tourist-like* and *Stowaway-like* MITE sub-groups (with 3 bp TAA and 2 bp TA TSDs, respectively) are derived from the *PIF/Harbinger-like* and *Tc1/mariner-like* elements, respectively (Stelmach et al., 2017). MITEs are mobilized by the transposase enzymes of their cognate autonomous Class II TEs, and thus are considered as truncated derivatives of these elements. MITEs tend to have higher copy numbers than their autonomous Class II TEs, which may be due to their lower *cis*-requirements for transposase recognition and/or the presence of enhancers for nucleoprotein complex formation within or near their TIRs (Dong et al., 2012; Macko-Podgórni et al., 2019; Tang et al., 2019). An example of this is the *Activator* (Ac) and *Dissociation* (Ds) elements in *Z. mays*, where Ac is an autonomous Class II TE and Ds is a non-autonomous Class II TE that can only transpose in the presence of Ac (Borlini et al., 2019).

The frequency and abundance of MITEs influence the structural diversity of their host genomes and the expression of host genes and phenotypes (Anderson et al., 2019; Suguiyama et al., 2019). This phenomenon has been documented in several plant species, such as mulberry (*Morus notabilis*) (Xin et al., 2019), grape (*Vitis vinifera*) (Benjak et al., 2009), and carrot (*Daucus carota*) (Macko-Podgórni et al., 2019). MITEs are enriched on chromosome arms and often associated with genes. For instance, MITE insertion into genes or regulatory regions alters gene expression and disrupts the vernalization requirement for flowering in *T. aestivum* (Yan et al., 2004). Thus, MITE-derived molecular markers are useful for gene tagging. MITEs are also frequently co-transcribed with plant genes. This is supported by the evidence that MITEs can provide coding sequences or poly(A) signals to genes and modulate the expression of host genes (Chen et al., 2012; Rohilla et al., 2022) Depending on the presence of regulatory motifs, MITEs may either increase or decrease gene expression (Han et al., 2016). MITE-derived microRNAs (miRNAs) regulate target gene expression at the transcriptional or post-transcriptional level. It was found that 6.5% of *Arabidopsis thaliana* and 35% of rice (*Oryza sativa*) miRNAs derive mainly from MITEs (He et al., 2015; Crescente et al., 2018). In *Solanaceae*, MITE-derived sRNAs are likely produced by the small interfering RNA biogenesis pathway (Kuang et al., 2009). These results indicate that MITEs have a significant role in both genome evolution and gene regulation.

Barley (*Hordeum vulgare*) is the fourth most cultivated cereal crop worldwide, after *Z. mays*, *O. sativa*, and *T. aestivum*. It represents one of the earliest crops domesticated by humans and possesses diverse applications in the brewing industry, animal feed, and human nutrition in specific regions (Schulte et al., 2009; Mascher et al., 2017; Li et al., 2023). Notably, barley exhibits superior adaptability to harsh environments compared to *T. aestivum*, hence making it a staple food in the Tibetan Plateau region of China (Petersen et al., 2013; Yao et al., 2022). The availability of an excellent barley reference genome (Morex V3) and pan-genome provides a valuable resource for future investigations in functional genomics and genome evolution (Jayakodi et al., 2020; Mascher et al., 2021). However, the diversity and evolutionary dynamics of MITEs in barley have yet to be explored. In this study, we conducted a comprehensive genome-wide survey of MITEs in the barley genome and assessed their amplification profile, impact on gene regulation, and evolutionary history. This study establishes a robust foundation for further elucidating the function and regulatory mechanisms of MITEs in barley.

## 2 Materials and methods

### 2.1 Identification of MITEs in barley

The barley Morex V3 reference assembly was obtained from the IPK database (http://doi.org/10.5447/ipk/2021/3). MITE candidates in the barley genome were identified using MITE Tracker with default parameters (Crescente et al., 2018; Riehl et al., 2022). The identified MITEs with the characteristic structure and parameter conditions were classified into distinct families by MITE Tracker. To facilitate multiple sequence alignment (MSA), MUSCLE v5.1 was employed for aligning the MITE sequences within each family (Edgar, 2022). In cases where MITEs lacked clear boundaries, we added 50 bp at both ends using custom Python scripts and repeated the MSA. Subsequently, consensus sequences with complete boundaries were generated using the WebLogo Tool (http://weblogo.berkeley.edu/logo.cgi). The classification of MITE families into superfamilies was based on the similarity of TIRs and TSDs sequences (**Supplementary Table S1**), and the annotation results were validated using DeepTE (Yan et al., 2020). Each MITE family was designated as HvX#, where Hv, X, and # represent *Hordeum vulgare*, the superfamily, and the family number, respectively. The superfamily designations T, P, h, M, C, and N corresponded to *Tc1*/*mariner-like*, *PIF*/*Harbinger-like*, *hAT-like*, *Mutator-like*, *CACTA-like*, and Unknown, respectively. A Python script was utilized to analyze the A/T base content and length of the identified MITEs. Additionally, the RNAfold web server (http://rna.tbi.univie.ac.at/cgi-bin/RNAWebSuite/RNAfold.cgi) was employed to predict the secondary structure of the superfamilies.

### 2.2 Genomic distribution of MITEs

The barley genome annotation file was acquired from the IPK database (http://doi.org/10.5447/ipk/2021/3). The relative positions between MITEs and genes were analyzed using BEDTools v2.30.0 (Quinlan and Hall, 2010). The MITE insertion region was categorized into intergenic region, gene region (including intron and exon), 5’ flanking region (upstream 5 kb), and 3’ flanking region (downstream 5 kb). In addition, to consider the presence of *cis*-regulatory elements in the promoter region, a 2 kb upstream region from the gene’s 5’ end was defined. Each MITE that intersected with any of these regions was counted as one insertion, even if it spanned multiple regions. The genomic distribution of genes and MITEs on each chromosome was visualized using the R package RIdeogram with a window size of 1 MB (Hao et al., 2020). To investigate the insertion preference of MITEs around genes, the 5’ flanking region (upstream 5 kb) and the 3’ flanking region (downstream 5 kb) were further divided into 10 equal segments, each spanning 500 bp. The resulting data were visualized using the ggplot2 package v3.5.1 in R to explore the correlation between the distribution of MITEs and their distance from genes. For functional annotation, Gene Ontology (GO) and Kyoto Encyclopedia of Genes and Genomes (KEGG) annotation were performed using eggNOG-mapper v2 (http://eggnog-mapper.embl.de/) (Cantalapiedra et al., 2021) with default parameters. Subsequently, GO term and KEGG pathway enrichment analyses were conducted using TBtools v1.129 (Chen et al., 2020a). Enrichment with Q-values ≤ 0.05 was considered statistically significant.

### 2.3 Expression level and tissue specificity analysis

A dataset of 96 RNA-seq samples was obtained from the National Center for Biotechnology Information (NCBI) Sequence Read Archive (SRA) database (PRJEB14349), encompassing 16 different barley tissues or stages (**Supplementary Table S2**). The SRA files were downloaded using the prefetch option in SRAToolkit v2.10.8 and subsequently converted into FASTQ files using the parallel-fastq-dump tool (https://github.com/rvalieris/parallel-fastq-dump). To ensure data quality, Trimmomatic v0.36 was employed for raw read quality assessment (Bolger et al., 2014). The high-quality reads were aligned to the barley reference genome (Morex V3) using HISAT v2.1.0 (Kim et al., 2015). Sorting of the resulting BAM files was conducted using SAMtools v1.3.1 (Li et al., 2009). StringTie v1.3.5 (Pertea et al., 2015) was utilized to calculate the fragments per kilobase of transcript per million mapped reads (FPKM), representing the expression levels of each gene. To evaluate the tissue specificity of genes, the τ index was employed, as described in a previous study (Yanai et al., 2005). The τ index was calculated using the following formula:


$$\tau =\frac{\sum_{i}^{N}\left(1-\frac{x_{i}}{x_{max}}\right)}{N-1}$$

In the formula, N represents the total number of tissues, X_i_ represents the mean FPKM value in tissue i, and X_max_ denotes the maximum FPKM value across all tissues. The resulting τ values ranged from 0 to 1, with τ = 1 indicating absolute specificity in a single tissue and τ = 0 indicating equal expression across all tissues.

### 2.4 Estimating MITE insertion time

The insertion time of the MITE element can be estimated by calculating the divergence rate between individual members and their consensus sequences (Jiang et al., 2016). To estimate the age of the MITE, MUSCLE v5.1 was employed to align the MITEs within each MITE family. The consensus sequences of the family were extracted using BioEdit (Alzohairy, 2011). The nucleotide substitution level (k) between each MITE and the consensus sequence was estimated using the Kimura 2-parameter distance method (Kimura, 1980). The age of the MITE was then calculated using the formula T = k/2r × 10^−6^, where T represents million years ago (Mya), and assuming a substitution rate (r) of 1.30 × 10^−8^.

### 2.5 Identification of long terminal repeat retrotransposons

LTR retrotransposons were identified by merging the results from LTRharvest genometools v1.6.2 (Ellinghaus et al., 2008) and LTR_FINDER v1.1 (Ou and Jiang, 2019) using LTR_retriever v2.9.9 (Ou and Jiang, 2018). LTRharvest v1.6.2 was selected for its higher sensitivity, while LTR_FINDER v1.1 exhibited a lower false-positive rate (Aroh and Halanych, 2021). LTR retrotransposon candidates with the TGCA motif were identified using specific parameters in LTRharvest: “-minlentltr 100, -maxlenltr 7000, -mintsd 4, -maxtsd 6, -similar 90, -vic 10, -seed 20, -motif TGCA, -motifmis 1”. Subsequently, both TGCA and non-TGCA motif candidates were identified using specific parameters in LTR_FINDER v1.1: “-w 2 -C -D 15000 -d 1000 -L 7000 -l 100 -p 20 -M 0.85 -harvest_out -size 1000000 -time 300”. To filter out false-positive LTR retrotransposon candidates identified by LTRharvest v1.6.2 and LTR_FINDER v1.1, LTR_retriever v2.9.9 was employed with default parameters. The categorized LTR retrotransposons were then analyzed using TEsorter v1.4.6 and the plant dataset from the REXdb database (http://repeatexplorer.org/) for lineage-level classification, specifying the parameters “-db rexdb-plant” (Neumann et al., 2019; Zhang et al., 2022).

The time of initial insertion for LTR retrotransposon candidates was estimated using the LTR_retriever package v2.9.9. The estimation was based on the calculation T = K/2μ, where T represents the insertion time, K is the divergence rate determined using the Jukes-Cantor model (K = − 3/4*ln (1-d*4/3)), and μ is the neutral mutation rate set at 1.3 × 10^−8^ mutations per base pair per year (Aroh and Halanych, 2021).

### 2.6 Identification of MITE-derived miRNAs

A total of 22 small RNA-seq BioProjects comprising 366 samples were obtained from the NCBI SRA database (**Supplementary Table S3**). The quality assessment of raw reads from each sample was conducted using FastQC v0.11.9 (https://www.bioinformatics.babraham.ac.uk/projects/fastqc). Trim Galore v0.6.10 was employed for quality control and adapter trimming (https://www.bioinformatics.babraham.ac.uk/projects/trim_galore/). Reads with a length ranging from 18 to 30 nucleotide (nt) were selected for subsequent analysis. The prediction of RNA secondary structure was performed using the ViennaRNA package v2.5.1 (http://www.tbi.univie.ac.at/~ivo/RNA/). High-quality reads were aligned against the Rfam database using Bowtie software v1.3.1 (Langmead et al., 2009). Reads that mapped to non-coding RNAs, such as tRNA, rRNA, snRNA, and snoRNA sequences in the Rfam database v.13.0, with ≤1 mismatch, were excluded to minimize annotation noise. The filtered sequences were then aligned with the barley genome. Known and novel miRNAs in each sample were predicted using miRDeep-P2 v1.1.4 (Kuang et al., 2019) with default parameters. To identify miRNAs derived from MITEs, overlapping regions between MITEs and miRNA precursors were detected using the intersect function of BEDTools v2.30.0.

### 2.7 Comparative genomic and syntenic analysis

To investigate the evolutionary history of barley MITEs, we obtained the following datasets from various sources. The barley pan-genome project (Jayakodi et al., 2020) provided data from one wild barley, 11 landraces, and eight cultivars, which were accessed from the IPK database (http://doi.org/10.5447/ipk/2020/24). The wild barley accessions EC-S1 and EC-N1 (Zhang et al., 2023) were obtained from the China National GeneBank Database (https://db.cngb.org/search/project/CNP0003286/). The wild barley accession OUH602 (Sato et al., 2021) was acquired from the Barley Bioresource Database (http://viewer.shigen.info/barley/download.php). Additionally, the barley cultivar assemblies Stirling V1 and Clipper V1 (Hu et al., 2023) were obtained from the Pawsey Supercomputing Centre (https://data.pawsey.org.au/public/?path=/wcga-pangenome/Australian_barley_genomes_raw_data). For comparative analysis, we included the sea barleygrass (*Hordeum marinum*) (Kuang et al., 2022) from the Genome WareHouse database at the China National Genomics Data Center with BioProject accession number PRJCA009391 (https://ngdc.cncb.ac.cn/gwh/Assembly/25443/show), as well as the *Triticeae* species *T. urartu* (Ling et al., 2018), *Aegilops speltoides* (Li L. F. et al., 2022), *T. durum* (Maccaferri et al., 2019), *Ae. Tauschii* (Luo et al., 2017), and *Secale cereale* (Rabanus-Wallace et al., 2021) from the NCBI (https://www.ncbi.nlm.nih.gov/) database. Furthermore, we included *Sorghum bicolor*, *O. sativa*, *Brachypodium distachyon*, and *Z. mays* from the Ensembl Plants database (https://plants.ensembl.org/index.html). To characterize MITEs across these accessions, we utilized MITE Tracker and followed the same workflow. To reveal syntenic relationships between MITEs and genes, we employed MCscan software (https://github.com/tanghaibao/jcvi/wiki/MCscan-(Python-version)), using Morex V3 as the reference genome.

### 2.8 Phylogenetic tree construction

The protein sequences of nine *Poaceae* species, namely *T. urartu*, *T. durum*, *T. aestivum*, *Ae. tauschii*, *S. cereale*, *S. bicolor*, *O. sativa*, *B. distachyon*, and *Z. mays*, were retrieved from Ensembl Plants (https://plants.ensembl.org/index.html) to construct the species tree. Considering the polyploid nature of *T. aestivum*, it was separated into the A, B, and D subgenomes, while *T. durum* was divided into the A and B subgenomes, respectively. Orthologous groups were determined using OrthoFinder v2.5.4 with the parameters “-M msa -S diamond” (Emms and Kelly, 2019). Poorly aligned regions were eliminated using trimAl v1.4.rev15 with the parameters “-fasta -gt 0.6 -cons 60” (Capella-Gutiérrez et al., 2009). Phylogenetic analyses were performed using raxmlHPC-PTHREADS from RAxML v.8.2.12 with the parameters “-m PROTGAMMAJTT -f a -p 123 -x 123 -# 100” (Stamatakis, 2014). Divergence time estimation was carried out using MCMCTree v4.10.7 and codeml, both of which are part of the PAML v4.10.7 (https://github.com/abacus-gene/paml). Calibration points for the divergence between *O. sativa* and *T. aestivum* (median time = 51.75 Mya) and between *S. bicolor* and *Z. mays* (median time = 11.20 Mya) were obtained from the TimeTree database (http://www.timetree.org).

We further investigated the evolutionary relationships among species based on MITE analysis. Syntenic MITEs from *H. vulgare* were extracted for *T. urartu*, *T. durum* (divided into the A and B subgenomes), *T. aestivum* (divided into the A, B, and D subgenomes), *Ae. tauschii*, *B. distachyon*, and *S. cereale*. MSA of the syntenic MITEs was performed using MUSCLE v5.1. The aligned syntenic MITEs were merged into the Phylip format and subjected to screening using the trimal v1.4.rev15 with default parameters. The species tree was constructed using raxmlHPC-PTHREADS from RAxML v.8.2.12 with the parameters “-m GTRGAMMA -f a -p 123 -x 123 -# 100”. Divergence time estimation was performed using MCMCTREE with the approximate likelihood method. The calibration time for the divergence was obtained from the TimeTree database, setting the median time between *O. sativa* and *T. aestivum* as 51.75 Mya.

## 3 Results

### 3.1 Mining and characterization of MITEs in the barley genome

**Figure 1:**
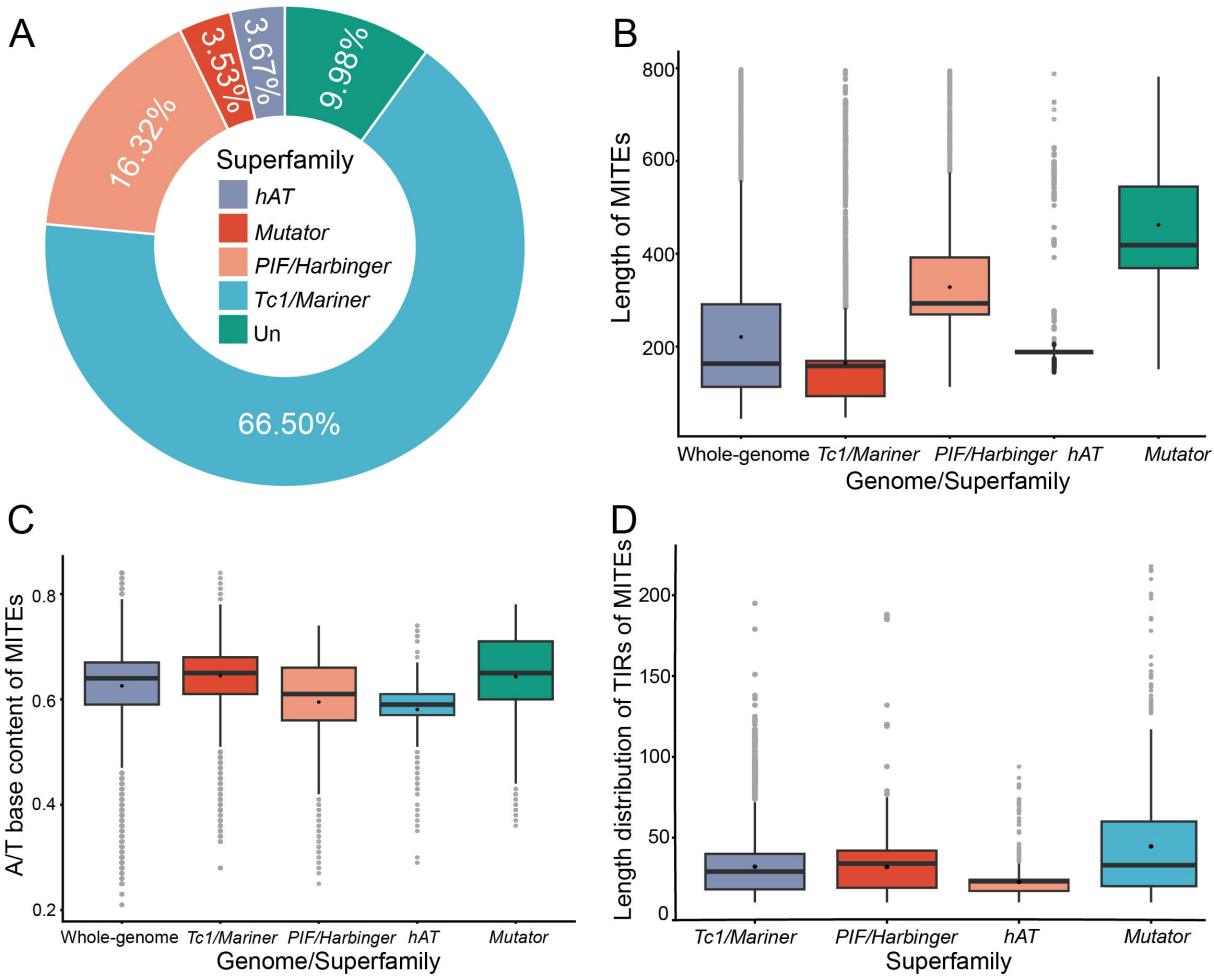
Characterization of MITEs in barley. **(A)** Number and proportion of MITE superfamilies, with “Un” representing unclassified MITEs. **(B)** Length distribution of MITEs. **(C)** Statistics on A/T bases content of MITEs. **(D)** Length distribution of TIRs of MITEs.

The MITE Tracker pipeline identified a total of 32,258 MITEs, comprising 30,171 unique MITEs (**Supplementary Tables S4**, **S5**). The total length of MITEs in the barley genome was 7.12 Mb, which accounted for only 0.17% of the genome. This finding suggests that MITEs may have a role in shaping the genomic structure of barley. The proportion of MITE sequences in barley was comparatively lower when juxtaposed with *O. sativa* and *S. bicolor* (Chen et al., 2014). However, the MITE content in barley was consistent with that observed in *T. aestivum* (Crescente et al., 2018). Interestingly, although large genomes are typically associated with the expansion of repetitive elements, there was no strong positive correlation between the proportion of MITEs and genome size in the host genome. Furthermore, the VSEARCH workflow dereplicated and clustered the MITEs into 2992 distinct families based on their similarities. The family size ranged from 3 to 1913 members, with an average of 11 members. Based on the sequence characteristics of TIRs and TSDs, MITE families were classified into four superfamilies (**Figure 1A**). *Tc1/Mariner-like* MITEs were the most abundant, comprising 21,450 MITEs in 2014 families (66.50%), followed by *PIF/Harbinger-like* MITEs with 5266 MITEs in 407 families (16.32%). In contrast, the *hAT-like* MITEs and *Mutator-like* MITEs were less abundant, with 1183 MITEs in 98 families (3.67%) and 1138 MITEs in 68 families (3.53%), respectively. The remaining 3221 MITEs in 405 families (9.98%) were unclassifiable and labeled as unkown. No *CACTA-like* MITEs were identified in barley, a phenomenon also observed in *M. notabilis and A. thaliana* (Guo et al., 2017; Xin et al., 2019). The distribution of MITEs in each superfamily varied significantly in barley, possibly related to the number and activity of the autonomous TEs corresponding to the distinct MITEs (He et al., 2015).

MITEs are characterized by their short sequence length. The length of MITEs in the barley genome ranges from 50 to 800 bp, with a mean of 220 bp (**Figure 1B**). Barley has more moderate length of MITEs than other crops, such as *O. sativa* (291 bp), *Z. mays* (329 bp), and *T. aestivum* (225 bp) (**Supplementary Table S6**). Analysis of different superfamilies of MITEs revealed that *PIF/Harbinger-like* MITEs (mean length 328 bp, coefficient of variation 37.14%) and *Mutator-like* MITEs (462 bp, 35.35%) were longer and more clustered, whereas *hAT-like* (215 bp, 46.70%) and *Tc1/Mariner-like* MITEs (164 bp, 65.40%) were shorter and more dispersed. MITE length differed significantly among different subfamilies (Mann-Whitney U-test, p<0.001).

MITEs are primarily AT-rich, have a propensity to integrate into AT-rich intergenic regions of the genome, and generate transcripts that result in stem-loop secondary structures that are thermodynamically stable (Minnick, 2024). They form secondary structures that stabilize them in a single-stranded state during transposition, possibly enhancing MITE transposition efficiency (Chen et al., 2012). The overall barley MITEs contained 61.61% A/T base content. The composition of A/T bases in four superfamilies was as follows: *Tc1/Mariner-like* MITEs (64.76%), *PIF/Harbinger-like* MITEs (58.96%), *hAT-like* MITEs (57.80%), and *Mutator-like* MITEs (58.72%) (**Figure 1C**). The *Tc1/Mariner-like* and *PIF/Harbinger-like* MITEs contained a greater proportion of A/T bases, which increased their tendency to form secondary structures in a single-stranded state, enhancing their insertion success rate and giving them a numerical advantage in the host genome. In contrast, *hAT-like* and *Mutator-like* MITEs contained a lower proportion of A/T bases, which reduced their likelihood of forming secondary structures in a single-stranded state, lowered their insertion success rate, and led to a lower content in the host genome. The A/T base content analysis results agreed with the MITE member number analysis results for each superfamily.

MITEs have TIRs at both ends, which enable them to form stem-loop secondary structures by self-complementary pairing in a single-stranded state (Milanowski et al., 2014). Our results indicated that the TIRs length of the same superfamily was relatively constant, suggesting the high conservation of their structure (**Figure 1D**). Moreover, the TIRs length was also closely correlated with the stem-loop length, which might affect the transposition efficiency and stability of MITEs (Chen et al., 2012). In barley, the secondary structures of different superfamilies showed significant variation. The AT-rich *Tc1/Mariner-like* superfamily members had simple secondary structures in a single-stranded state, displaying typical intermediate stem complementary structures with multiple loops at both ends (**Supplementary Figure S1A**). In contrast, the *PIF/Harbinger-like* superfamily members tended to form multiple loop structures, which reduced the stability of their secondary structures (**Supplementary Figure S1B**). The secondary structures of MITE members from other superfamilies, such as *hAT-like* and *Mutator-like*, exhibited greater complexity and lower structural stability (**Supplementary Figures S1C, D**).

### 3.2 Genomic distribution of MITEs

**Figure 2:**
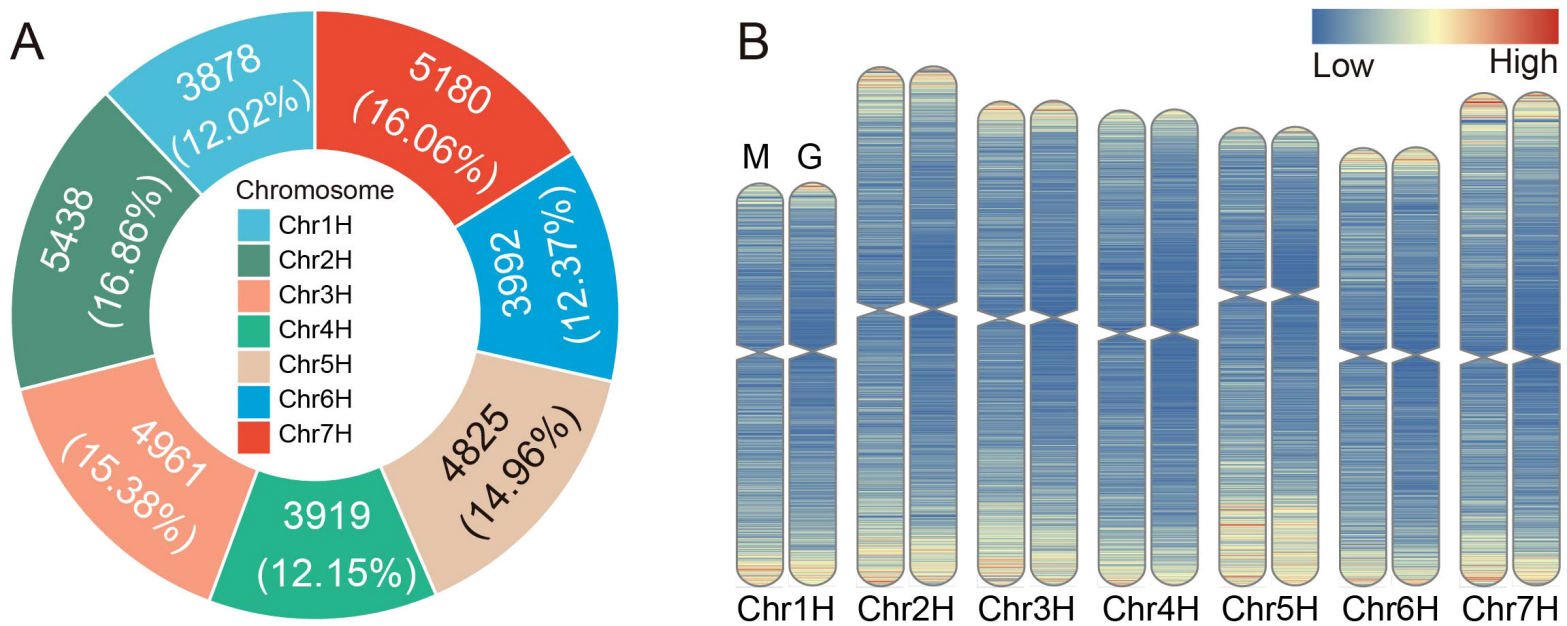
Distribution of MITEs on the barley chromosomes. **(A)** Number and proportion of MITEs on each chromosome. **(B)** Comparison of density distribution between genes and MITEs. Left (M): Density distribution of MITEs on the chromosome. Right (G): Density distribution of genes on the chromosome. Increasing densities are represented by a color gradient from blue to red.

The chromosomal density profile analysis revealed an uneven distribution of MITEs across different chromosomes in barley (**Figure 2A**; **Supplementary Table S7**). Significant correlation analysis indicated that longer chromosomes tended to harbor a higher number of MITEs (*p*-values ≤ 0.05). Among them, the highest number of MITEs was observed on chromosome 2H (5438, accounting for 16.86%), while the lowest number was found on chromosome 1H (3878, accounting for 12.02%). Our findings demonstrated that MITE transposons in the barley genome preferentially inserted into the pericentromeric regions of chromosomes, exhibiting a higher density in these regions. Conversely, the centromeric regions of chromosomes, which are compact and highly condensed, posed challenges for MITE insertion (**Figure 2B**), and the barley pan-genomic MITE also showed a similar distribution on chromosomes (**Supplementary Figure S2**). Furthermore, MITE transposons showed a tendency to insert into gene-rich regions, thereby potentially affecting the expression and functionality of host genes.

**Figure 3:**
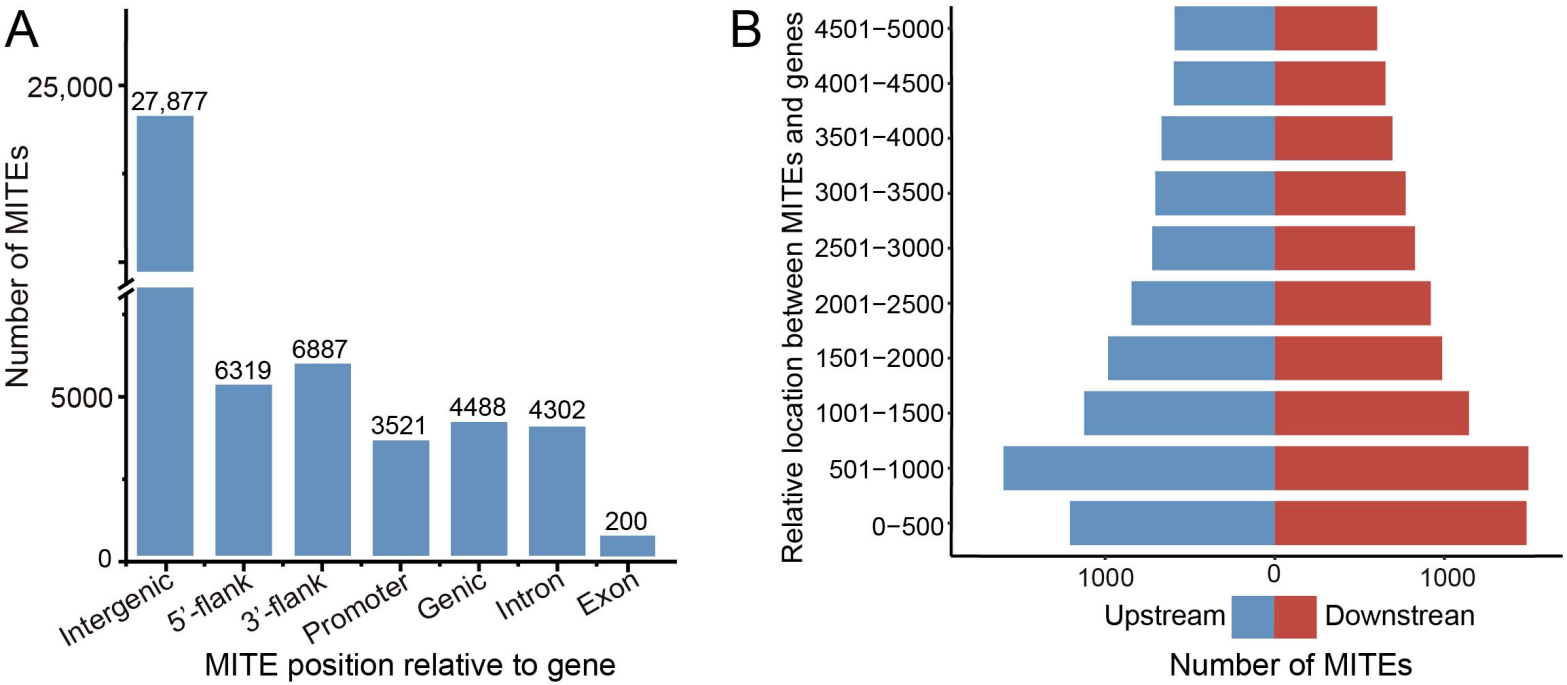
MITE genomic location statistic. **(A)** Distribution of MITE insertions across the barley genome. The terms “5’ flank” and “3’ flank” refer to the 5’ flanking region (upstream 5k bp) and the 3’ flanking region (downstream 5k bp) of the gene, respectively. The term “Promoter” donates the upstream 2k bp region. **(B)** Number of MITE insertions near genes in barley. The blue color bar represents MITE distribution in the 5’ flanking region (upstream 5k bp), while the red color bar represents MITE distribution in the 3’ flanking region (downstream 5k bp).

We conducted a systematic analysis of the distribution of MITEs across different genomic regions. The relative abundance of MITEs was calculated in the intergenic regions, 5’ flanking regions (upstream 5 kb), 3’ flanking regions (downstream 5 kb), and genic regions (including exons and introns). The results showed that the majority (27,877, 86.42%) of MITE insertions were concentrated in the intergenic regions (**Figure 3A**). We defined a MITE insertion within 5 kb upstream or downstream of a gene as a near-gene region. In the barley genome, there were 13,206 MITEs inserted in the near-gene regions, specifically, 6319 MITE insertions involved the 5’ flanking regions and 6687 MITE insertions involved the 3’ flanking regions, with slightly more MITEs in the 3’ flanking regions than in the 5’ flanking regions. Considering that the promoter regions contain abundant *cis*-elements that interact with RNA polymerase and transcription factors to regulate the timing and level of gene expression, we paid extra attention to the 2 kb upstream promoter regions of the genes. The results showed that 3521 MITE insertions were associated with the promoter regions, accounting for about 55.72% of the MITEs in the 5’ flanking regions. In addition, we found that 4488 MITEs (13.91% of the total MITEs) were inserted into 3479 genes (9.71% of the total genes). Among them, 4302 MITEs were inserted into intron regions, and only 200 MITEs were inserted into exon regions.

To investigate the preferential insertion of MITEs flanking genes, we divided the gene flanking regions into 10 equal segments of 5 kb each (500 bp per segment) (**Figure 3B**). Analysis of MITE insertion preferences in each segment revealed a higher frequency of insertions as the distance to the gene decreased. At the 5’ end of the gene, the number of transposon insertions gradually increased, reaching a peak in the 501–1000 bp region (1598 insertions), while the closest 0-500 bp segment had slightly fewer insertions (1207). This suggests that regions within 5 kb of the 5’ end of the gene experience lower negative selection pressure and inhibition, leading to more frequent MITE activity. A similar trend was observed at the 3’ end of the gene, with a peak in the 501–1000 bp region (1494 insertions) and no significant drop in the closest 0-500 bp segment.

Distinct subfamilies of MITEs exhibited noticeable variations in their insertion patterns within the genome. For instance, the *PIF/Harbinger-like* MITE family showed a higher propensity for insertions in proximity to genes, with 16.47% and 15.53% of insertions occurring in the 5 kb upstream and downstream regions, respectively. In contrast, the *Tc1/Mariner-like* MITE family exhibited a significantly higher insertion rate (13.02%) within genic regions compared to other families. The *Mutator-like* and *hAT-like* elements displayed a stronger preference for intergenic regions, with infrequent insertions in introns and negligible insertions in untranslated regions and exons (**Supplementary Table S8**).

### 3.3 MITE insertion on genome structure and gene expression

**Figure 4:**
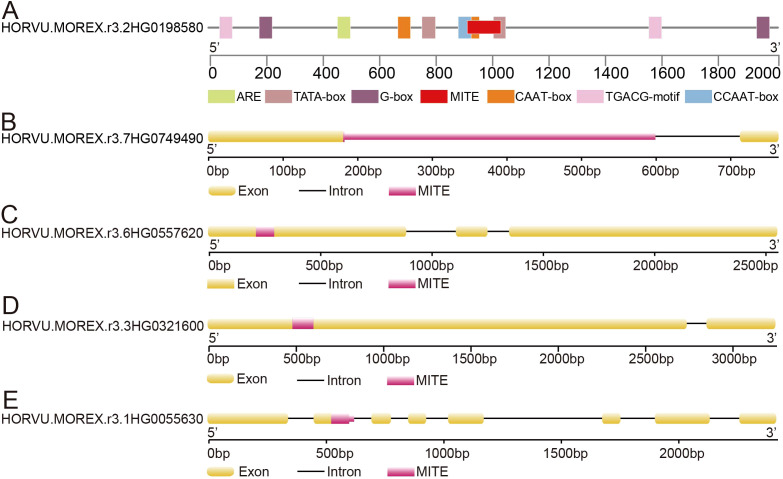
The impact of MITE insertion on gene structure. **(A)** MITE insertion within the gene’s promoter region. **(B)** MITE insertion within the gene’s intron region. **(C, D)** MITE insertion within the gene’s exon region. **(E)** MITE insertion spanning the intron-exon junction of the gene.

Our findings demonstrate that the majority of MITE inserted into the flanking regions of genes, while a smaller fraction inserted within near-gene regions and gene bodies (**Supplementary Table S9**). These insertions had a significant impact on gene expression regulation and even resulted in alterations to the original gene structure, ultimately leading to the termination of normal gene expression. For instance, we identified a MITE insertion from the *Tc1/Mariner-like* family located at a distance of 904 bp upstream of the gene transcription start site in the *HORVU.MOREX.r3.2HG0198580* gene. The inserted sequence contained *cis*-regulatory elements, such as CCAAT-box, CAAT-box and TATA-box (**Figure 4A**), which are associated with biological pathways related to plant growth, development, and responses to stress conditions. Additionally, the *PIF/Harbinger-like* superfamily of MITE inserted into the intronic region of the *HORVU.MOREX.r3.7HG0749490* gene, resulting in a substantial increase in gene length (**Figure 4B**). Moreover, within the first exon of the *HORVU.MOREX.r3.6HG0557620* gene, a MITE insertion from the *Tc1/Mariner-like* family, spanning 81 bp, caused a frameshift mutation in the original gene sequence (**Figure 4C**). Similarly, the *Tc1/Mariner-like* family of MITEs inserted into the first exon of the *HORVU.MOREX.r3.3HG0321600* gene, leading to an increase of 119 bp in the gene length (**Figure 4D**). We also identified MITE insertions spanning intron-exon boundaries, which potentially influenced gene alternative splicing (**Figure 4E**). Collectively, these examples underscore the significant role of MITEs in driving structural variations in the barley genome.

RNA-seq datasets from 16 distinct tissues and stages of barley were analyzed to identify potential genes influenced by MITE insertions. Gene expression levels were quantified using FPKM, and a tissue specificity index was calculated. We identified 91 MITE insertions ranging in length from 82 to 699 bp, predominantly located in gene promoter regions. The downstream genes associated with these insertions exhibited highly specific expression patterns in different tissues, indicated by a τ value of 1. Notably, among these insertions, 67 MITEs (accounting for 73.63% of the total inserted MITEs) belonged to the *Tc1/Mariner-like* superfamily (**Supplementary Table S10**). These findings provide promising candidates for further experimental investigations.

**Figure 5:**
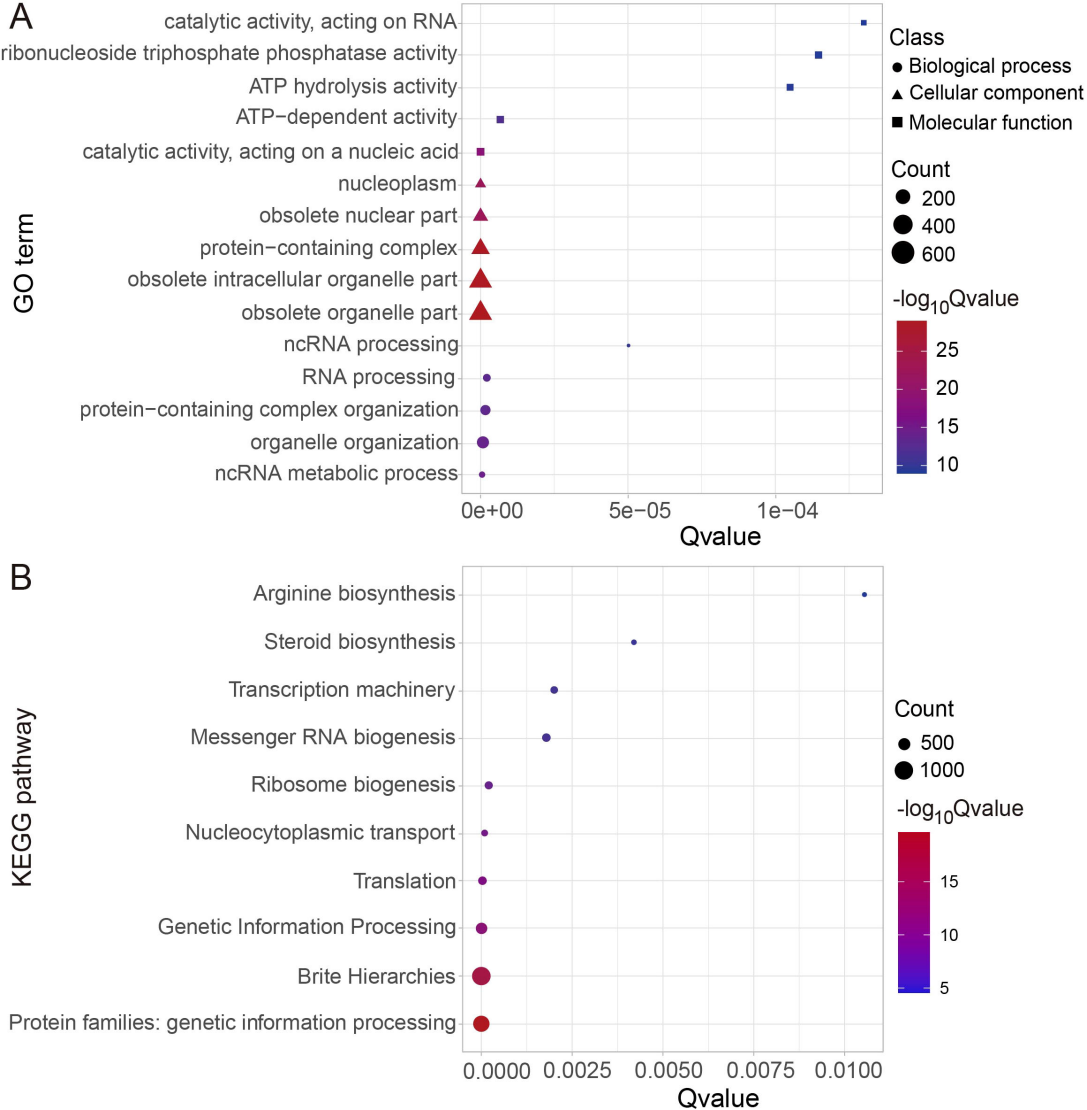
Functional enrichment analysis of MITE-related genes. **(A)** GO enrichment analysis of MITE-related genes. **(B)** KEGG pathway enrichment analysis of MITE-related genes.

To elucidate the biological functions of genes affected by MITE insertions, we conducted GO enrichment analysis (**Figure 5A**; **Supplementary Table S11**). In the major categories of the biological process, significant gene enrichment was observed in organelle organization (GO:0006996), protein-containing complex organization (GO:0043933), and RNA processing (GO:0006396). Regarding cellular components, the genes were primarily associated with functions in the obsolete organelle part (GO:0044422), obsolete intracellular organelle part (GO:0044446), and protein-containing complex (GO:0032991). Furthermore, they were enriched in ribonucleoside triphosphate phosphatase activity (GO:0017111), ATP hydrolysis activity (GO:0016887), and ATP-dependent activity (GO:0140657) in the molecular function category. Additionally, we performed KEGG pathway enrichment analysis for these genes, revealing their involvement in genetic information processing (KO09182 and KO09120), brite hierarchies (KO09180), translation (KO09122), nucleocytoplasmic transport (KO03013), ribosome biogenesis (KO03009), messenger RNA biogenesis (KO03019), transcription machinery (KO03021), steroid biosynthesis (KO00100), and arginine biosynthesis (KO00220) (**Figure 5B**; **Supplementary Table S12**).

### 3.4 LTR retrotransposons characterization, classification and annotation

LTR retrotransposons are the most abundant TEs in plant genomes (Park et al., 2021). In this study, we employed an integrated approach to identify LTR retrotransposons in the barley genome and compare their distribution with that of MITEs. A total of 45,710 intact LTR retrotransposons were identified and classified into two main categories: *Copia-like* elements (22,210, 48.59%) and *Gypsy-like* elements (22,085, 48.32%) (**Supplementary Figure S3A**). LTR retrotransposons that did not fit into these categories (1415, 3.10%) were classified as Unknown and excluded from subsequent analysis. The insertion locations of LTR retrotransposons exhibited a similar pattern to that of MITEs. The majority of intact LTR retrotransposons (44,178) were found in intergenic regions, followed by 983 LTR retrotransposons in gene regions. Among these, 671 LTR retrotransposons (82.71% from *Copia-like* elements) were located within introns, while 584 (58.27% from *Gypsy-like* elements) were present in exons. Additionally, 2704 LTR retrotransposons were inserted upstream of genes, and 2403 were inserted downstream within 5 kb regions. Notably, 1177 LTR retrotransposons were inserted into the promoter region (2 kb upstream) (**Supplementary Figure S3B**).

By analyzing the distribution of LTR retrotransposons in the barley genome, we observed a notable contrast to the relatively uniform genomic distribution of MITEs. Specifically, the distribution of LTR retrotransposons exhibited significant heterogeneity. Consistent with previous investigations in other species (Du et al., 2010; Liu et al., 2019), we found a substantial enrichment of LTR retrotransposons in regions proximal to the centromeres across different chromosomes (**Supplementary Figure S3C**). This intriguing observation can potentially be attributed to the recombination-suppressed nature of centromere-proximal regions. The suppression of unequal homology recombination and illegitimate recombination in these regions may lead to the accumulation of LTR retrotransposons.

### 3.5 Insertion time estimation

**Figure 6:**
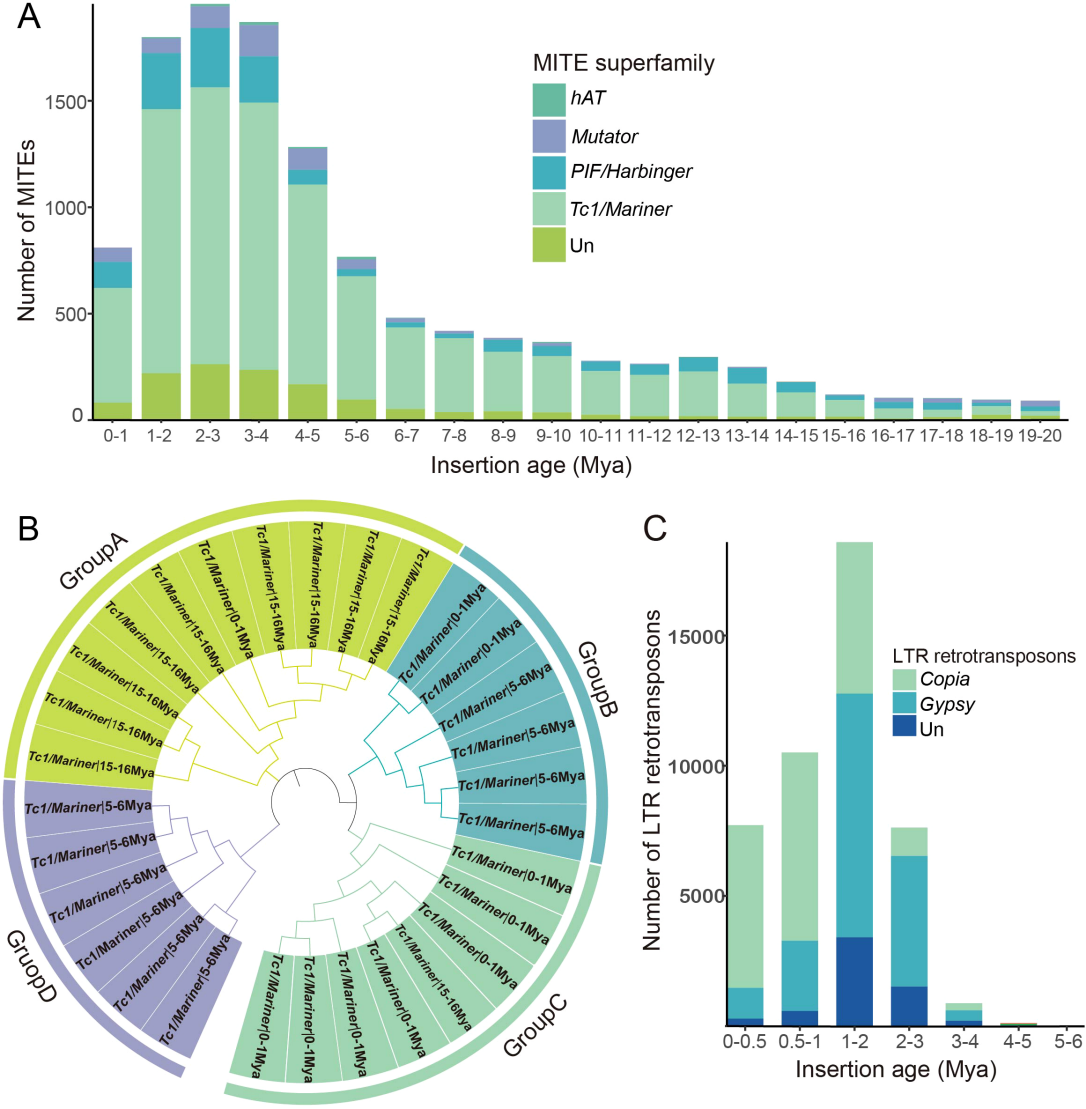
Temporal dynamics of MITE and LTR retrotransposon insertion events. **(A)** Timeline of MITE insertions, highlighting transpositional “bursts” depicted by peaks, with distinct transposon superfamilies color-coded across phases. **(B)** Cluster analysis of partial sequences originating from the *Tc1/Mariner-like* superfamily at three specific time intervals: 0-1 Mya, 5-6 Mya, and 15-16 Mya. **(C)** The timeline of LTR retrotransposon insertions.

The divergence rate between individual members and their consensus sequences can be utilized to estimate the age of TEs (Jiang et al., 2016). Our analysis revealed that a large proportion of barley MITEs were inserted in the recent 20 million years, with a significant proportion inserted within the last 5 million years. The insertion patterns of barley MITEs exhibited two notable peaks. A smaller-scale peak occurred around 12-13 Mya, involving 297 MITEs mainly from the *PIF/Harbinger-like* and *Tc1/Mariner-like* superfamilies. Another intense expansion occurred around 2-3 Mya, with more than 1955 MITEs participating from the superfamilies *PIF/Harbinger-like*, *Tc1/Mariner-like*, *Mutator-like*, and *hAT-like*. These superfamilies displayed similar bimodal patterns, indicating two distinct amplification events that coincided with the overall MITE expansion timing. Notably, the *Tc1/Mariner-like* MITEs exhibited a higher insertion rate and dominated the transposition explosion, followed by *PIF/Harbinger-like* MITEs, while the *hAT-like* and *Mutator-like* superfamilies had low participation, consistent with their lower member numbers (**Figure 6A**). Clustering analysis of MITEs from different amplification nodes revealed that MITEs with similar amplification times tended to be closer in evolutionary relationships (**Figure 6B**).

Similarly, LTR retrotransposons undergo constant insertion and elimination in a long-term cycle, maintaining a dynamic balance in the host genome size. We determined the insertion time of LTR retrotransposons, and their burst occurred within a concentrated period approximately 1-2 Mya, involving 18,585 LTR retrotransposons (5817 *Copia-like*, 9358 *Gypsy-like*) (**Figure 6C**). These findings indicate that LTR retrotransposons were active in a more recent and traceable past compared to MITEs, which is consistent with previous studies (Liu et al., 2019).

### 3.6 miRNAs derived from MITEs in barley

**Figure 7:**
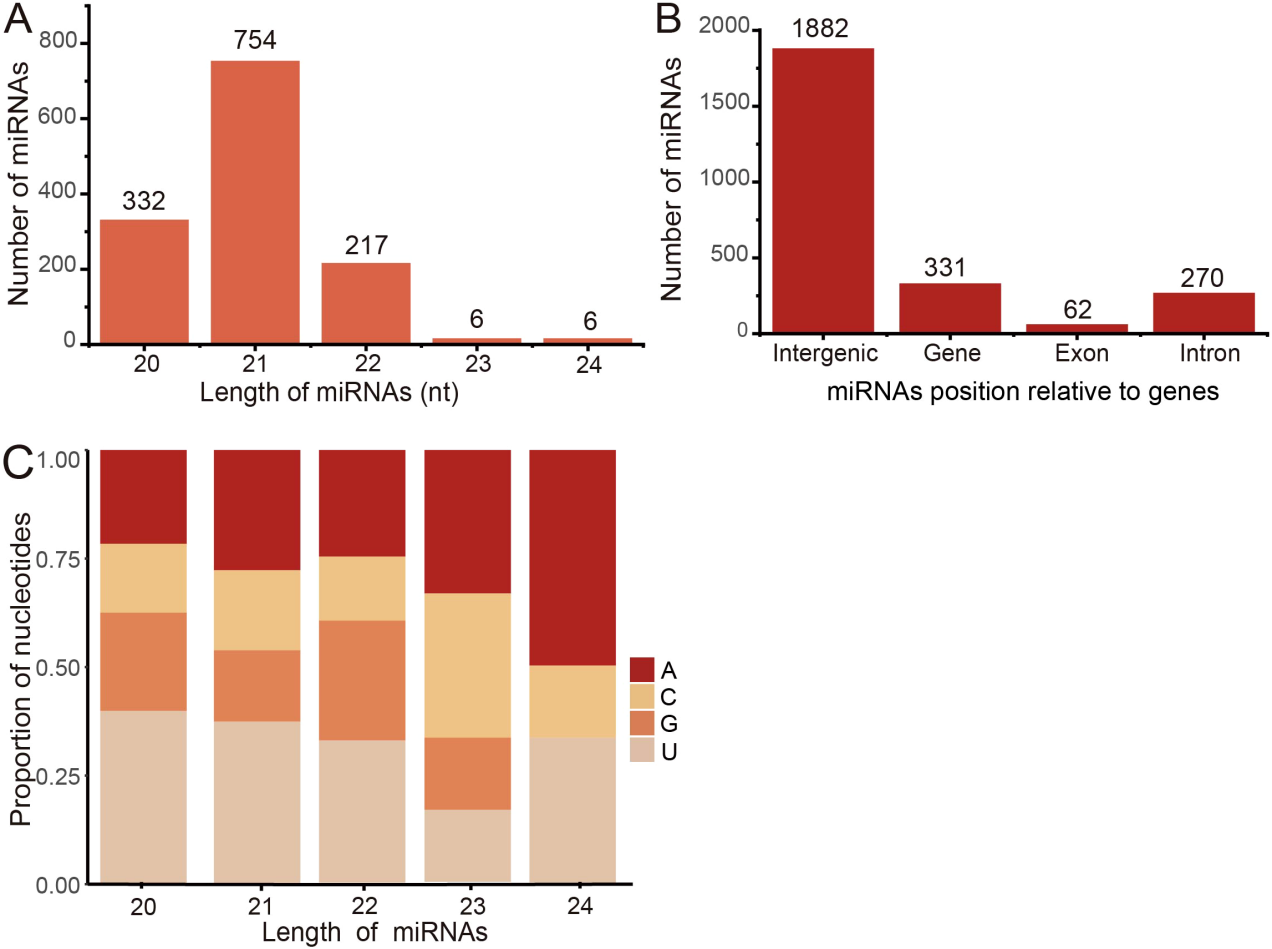
Characterization of miRNAs in barley. **(A)** The distribution of reads along with mature miRNA length. **(B)** Number of miRNAs at each genomic position. **(C)** Nucleotide bias of miRNAs at each position along the length of mature miRNAs.

We collected sequencing samples from 366 sRNAs across 22 BioProjects to establish a comprehensive collection of miRNAs. Utilizing the miRDeep-P2 pipeline, we identified a total of 1907 miRNA gene loci, which encoded a total of 2213 mature miRNAs (1315 non-redundant mature miRNAs). The length distribution analysis showed that the majority of miRNAs (754 non-redundant mature miRNAs, 57.34%) were predominantly 21 nt in length, followed by 20 nt (332, 25.25%) and 22 nt (217, 16.50%) (**Figure 7A**). Investigating their genomic distribution, we found that most miRNAs were located in intergenic regions (1882 redundant mature miRNAs, 85.04%), with a smaller proportion found within genic regions (331, 14.96%) (**Figure 7B**). Furthermore, 62 miRNAs (18.73%) were identified in exonic regions, 269 (81.27%) in intronic regions, and one miRNA (0.30%) spanning both exonic and intronic regions.

The distribution of miRNA gene loci across the seven barley chromosomes displayed unevenness, with chromosome 7H harboring the highest number of loci (381 miRNA gene loci, 17.22%). Conversely, the fewest miRNAs were observed on chromosome 4H (186, 8.40%). Notably, no significant correlation was found between the number of miRNAs and chromosome length (*p*-value ≥ 0.05), indicating that longer chromosomes did not necessarily contain a greater abundance of miRNAs. Previous studies have highlighted the influence of nucleotide composition on the physicochemical and biological properties of miRNAs, including their secondary structures (Feng et al., 2017). Considering the relatively low number of miRNA members with lengths of 23 nt and 24 nt, we focused our attention on miRNAs with lengths of 20 nt, 21 nt, and 22 nt. We observed a slight bias towards higher U content in their sequences (**Figure 7C**), which may play a crucial role in miRNA biogenesis and mRNA target recognition (Wang et al., 2015).

We further conducted a comprehensive investigation of miRNAs originating from MITEs. A total of 171 miRNAs derived from MITEs were identified, constituting approximately 7.73% of the total miRNAs. Among these, 152 miRNAs belonged to the *Tc1/Mariner-like* superfamily, 16 miRNAs to the *PIF/Harbinger-like* superfamily, and 3 miRNAs to the Unknown superfamily. The majority of MITE-derived miRNAs (98, 57.31%) were located in intergenic regions, while a smaller proportion was found within introns (68, 39.77%) and exons (5, 2.92%). Notably, our findings align with previous studies suggesting that LTR retrotransposons may also serve as a potential source of miRNAs (Guo et al., 2022). Specifically, we identified 11 miRNAs (0.49%) derived from LTR retrotransposons belonging to the *Copia-like* superfamily, further reinforcing the significance of MITEs as a valuable reservoir of miRNAs.

To investigate the tissue-specific expression of the 171 MITE-derived mature miRNAs, we measured their expression levels across ten samples (PRJNA823894) (**Supplementary Figure S4** and **Supplementary Table S13**). Our analysis revealed distinct spatiotemporal expression patterns of these miRNAs, implying their potential importance in the growth and development of barley.

### 3.7 Evolutionary analysis of MITEs

To elucidate the evolutionary history of barley MITEs, we employed a standardized analysis pipeline to identify MITEs in eleven other *Poaceae* species (**Supplementary Table S14**). Among these species, *T. aestivum* possessed the highest number of MITEs (136,982 MITEs and 9203 families), followed by *T. durum* (102,250 MITEs and 6299 families), primarily due to their allopolyploid genome characteristics. On the other hand, *B. distachyon* (9448 MITEs and 951 families) and *O. sativa* (17,606 MITEs and 1674 families) exhibited the lowest number of MITEs.

**Figure 8:**
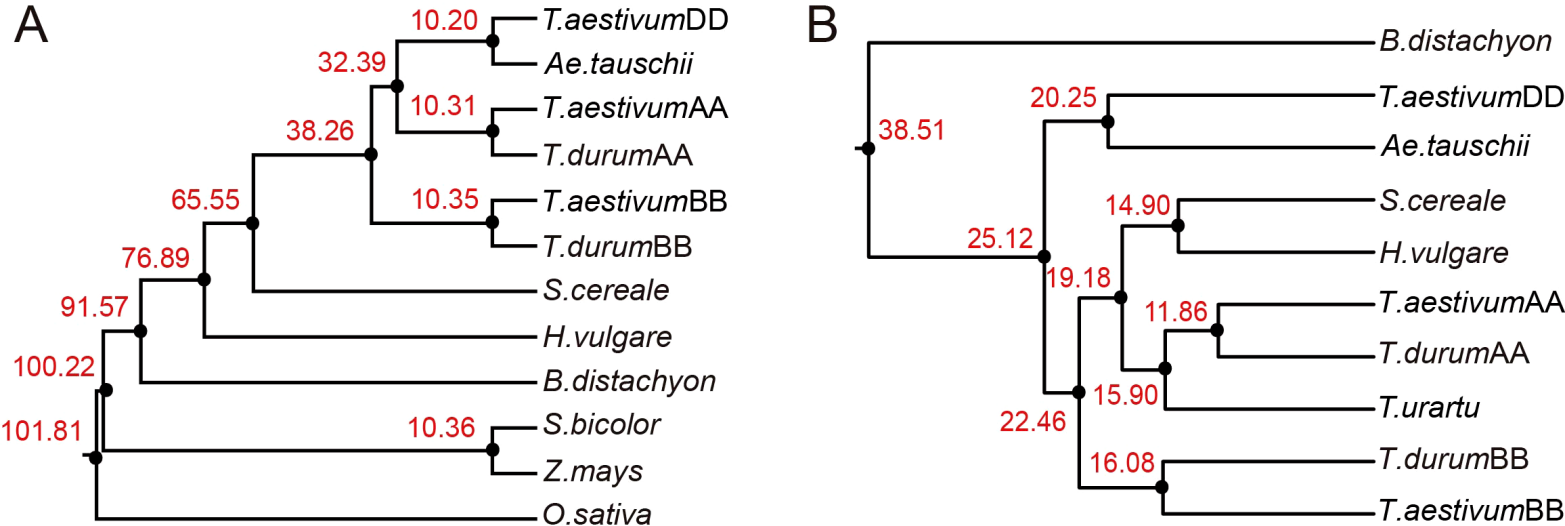
Phylogenetic analysis of species using genes and MITEs. **(A)** Phylogenetic trees and divergence times for nine *Poaceae* species based on orthologous genes. **(B)** Phylogenetic trees and divergence times of seven *Poaceae* species based on syntenic MITEs.

Based on MITE element analysis, collinearity assessment revealed that *H. marinum* and *Ae. speltoides* exhibited the highest collinearity ratios with barley, with values of 46.44% and 44.44% respectively. Conversely, *Z. mays* (0.05%), *S. bicolor* (0.14%), and *O. sativa* (0.65%) displayed the lowest collinearity ratios, which was consistent with the phylogenetic relationships among these species. Additionally, a gene-based collinearity analysis was performed, demonstrating higher gene collinearity ratios compared to MITE collinearity ratios. This observation suggests that genes were more conserved than MITEs. The phylogenetic tree constructed based on single-copy genes (**Figure 8A**), consistent with the phylogenetic tree based on conserved MITEs demonstrated the close relationship between barley and *S. cereale* (**Figure 8B**), implying a potential co-evolution of genes and MITEs during the evolutionary process.

We categorized MITEs occurring in all species as conserved MITEs, while those found only in certain species were classified as non-conserved MITEs. In barley, we identified a total of 1526 conserved MITEs and 10,154 non-conserved MITEs. Furthermore, our analysis demonstrated that the proportion of conserved MITEs inserted into promoters (0.16%) and 5’ and 3’ untranslated regions (0.03%) was lower compared to non-conserved MITEs (promoters: 1.15%, UTR: 0.14%). These findings indicate a strong selective effect of MITE insertion in these regions.

To investigate the evolutionary trajectory of MITEs during barley domestication, we analyzed the chromosome-level genomes of barley from various sources, including 4 wild barley accessions, 11 landraces, and 9 cultivars (**Supplementary Table S15**). Among these, the recently released genomes of wild barley EC-S1 (31,942) and EC-N1 (31,899) exhibited a slightly higher number of MITEs compared to other assemblies, utilizing the latest third-generation sequencing technologies. This finding highlights the superior capability of long-read sequencing technology in accurately detecting repetitive elements (Jayakodi et al., 2020; Mascher et al., 2021). Furthermore, our analysis revealed that the average number of MITEs in wild barley and cultivated barley was 31,243.25 and 30,579.55, respectively. This suggests that artificial selection during the domestication process may have led to the elimination of a small fraction of MITEs. When using Morex as a reference, the mean collinearity proportions of MITEs in wild barley, landraces, and cultivars were found to be 16.55%, 25.66%, and 27.71%, respectively. Similarly, collinearity proportions based on genes were higher than those based on MITEs, and there was a progressive increase in collinearity between wild barley (70.03%) and cultivated barley (73.34%) with the reference genome (Morex).

**Figure 9:**
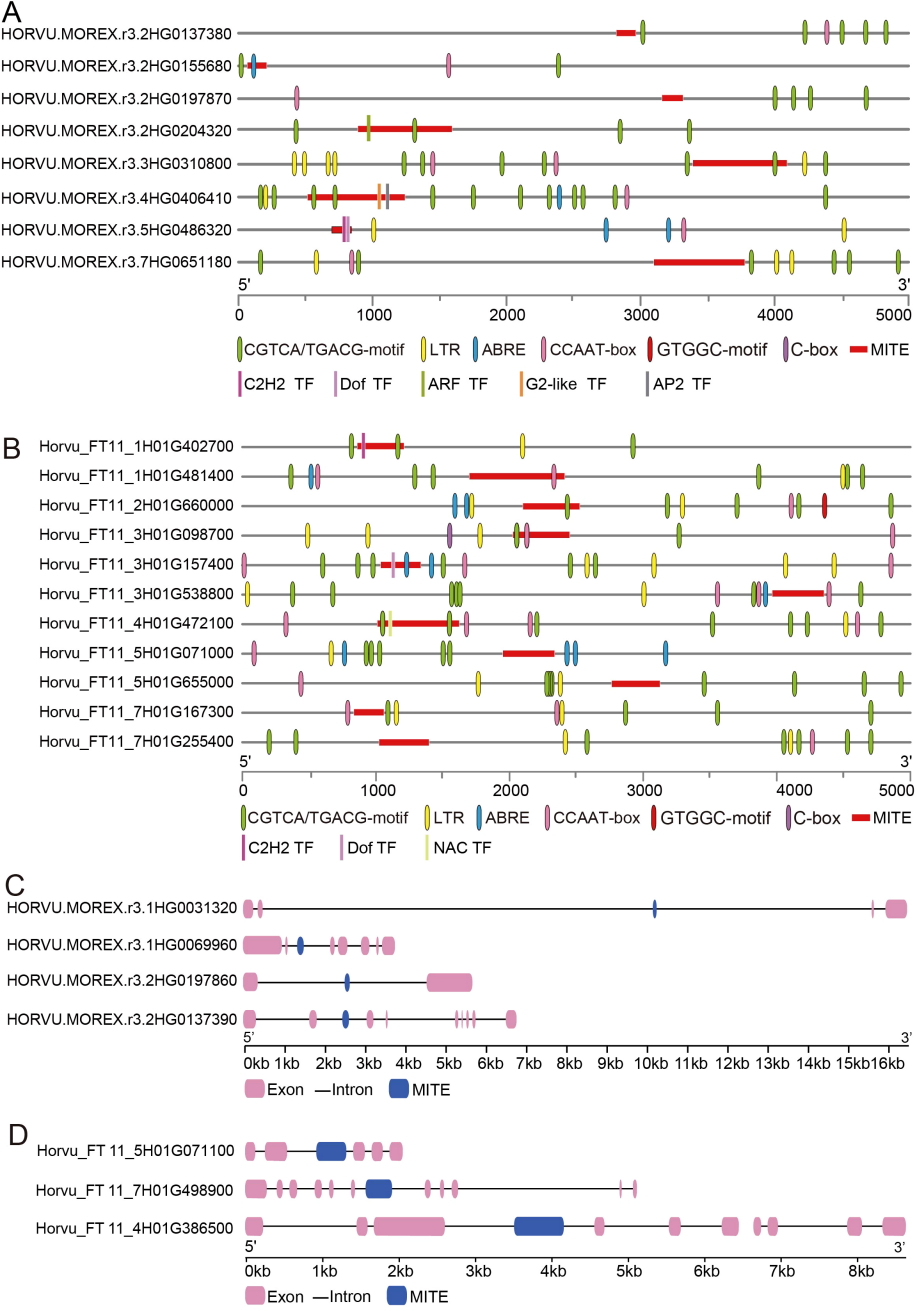
MITE dynamics throughout the barley domestication process. **(A)** MITE insertions within the promoter region. **(B)** MITE deletions within the promoter region. **(C)** MITE insertions within the genic region. **(D)** MITE deletions within the genic region.

In order to elucidate MITEs associated with barley domestication, we defined MITEs that are present in all cultivated barley varieties and absent in all wild barley accessions as domestication-inserted MITEs, while domestication-lost MITEs refer to those absent in cultivated barley varieties and present in wild barley accessions. Gene regulation has primarily been attributed to *cis*-elements in gene promoter regions. Therefore, we specifically focused on MITE insertions/deletions in these regions. We identified eight domestication-inserted candidate MITEs, such as MITEs inserted into the upstream 2kb region of the genes *HORVU.MOREX.r3.2HG0155680* (An unannotated gene), *HORVU.MOREX.r3.2HG0204320* (*ARF*), and *HORVU.MOREX.r3.4HG0406410* (*AP2*). One MITE was found in the promoter region of the *HORVU.MOREX.r3.2HG0155680* gene, harboring an ABRE *cis*-acting element associated with abscisic acid response. MITEs inserted into the promoter regions of the *HORVU.MOREX.r3.2HG0204320* and *HORVU.MOREX.r3.4HG0406410* genes contained a CGTCA/TGACG-motif linked to the methyl jasmonate response (**Figure 9A**; **Supplementary Table S16**). Additionally, we identified 11 MITEs that were lost during barley domestication. Among these, three MITEs were located in the upstream promoter regions of the genes *Horvu_FT11_1H01G402700* (*C2H2*), *Horvu_FT11_3H01G157400* (*Dof*), and *Horvu_FT11_4H01G472100* (*NAC*) in the wild barley accession BIK-04-12. Notably, the promoter regions of the *Horvu_FT11_1H01G402700* and *Horvu_FT11_3H01G472100* genes exhibited deletions of the CGTCA/TGACG-motif, while the promoter region of the *Horvu_FT11_3H01G157400* gene lacked the ABRE *cis*-acting element (**Figure 9B**). In addition, we identified domestication-associated MITE elements occurring within intronic regions. For example, during the domestication of barley, we observed a 161 bp MITE insertion (*Tc1/Mariner-like* family) within the second intron of *HORVU.MOREX.r3.1HG0069960* (EF-hand family) (**Figure 9C**). Conversely, the sixth intron of the wild barley gene *Horvu_FT11_7H01G498900*, hosting a CRAL/TRIO domain, encountered a 346 bp MITE deletion (**Figure 9D**). We hypothesize that these intronic MITE insertions or deletions might influence gene expression or alter splicing patterns.

## 4 Discussion

MITE-induced polymorphisms confer novel genomic diversity, potentially aiding host organisms in adapting to environmental changes, particularly stresses (Hou et al., 2021). Previous studies have demonstrated significant variation in the number of MITEs across species, which still correlates with genome assembly size. For example, *Glycine max* (973.34 Mb) harbors 126 MITE families comprising 169,379 MITEs, and *Z. mays* has a relatively larger genome (2058.58 Mb) with 252 MITE families containing 192,529 MITEs (Chen et al., 2014). Taking the Morex reference genome as an example, we identified 2,992 MITE families with 32,258 MITE-related sequences, which is reasonable considering the approximate 5 Gb genome size of barley. It is worth noting that existing tools for detecting hidden MITEs in genomes employ different methods and filtering criteria. MITE Tracker stands out by utilizing a fast and memory-efficient algorithm to identify potential MITEs in genome sequences. Additionally, its meticulous false-positive filtering criterion makes it the most accurate tool available (Crescente et al., 2018). With the inclusion of different barley accessions, the approximate 30,000 MITEs in barley account for 0.17% of the genome, which aligns with the findings in *T. aestivum*, a close relative of barley, where 0.16% of the *T. aestivum* reference genome is covered by MITEs (Crescente et al., 2018). These MITE fragments not only contribute information to the genome, but are also a source of diversity between varieties. It is noteworthy that various regions of the barley genome contain a considerable number of MITE insertions, indicating the wide distribution of MITE transposons and their potential as molecular markers.

MITEs preferentially distribute in gene-associated regions, potentially causing variations in host gene expression profiles under specific biological or abiotic stresses. Our analysis revealed a widespread distribution of MITEs throughout the barley genome, with a clear preference for regions characterized by high gene density, which is consistent with findings in other higher organisms (Zhou et al., 2016; Liu et al., 2019; Xin et al., 2019). A higher abundance of barley MITEs both upstream and downstream of the nearest genes compared to more distal regions were also observed. This distribution pattern suggests the rapid elimination of MITE insertions in intergenic regions from populations due to their deleterious effects. Notably, the substantial number of barley MITE insertions upstream of the nearest genes suggests that MITEs play significant roles in gene expression by altering regulatory motifs.

Given their high copy numbers, it is highly likely that additional MITEs within gene regions have functional implications, such as providing regulatory sequences or recruiting epigenetic modifications. For instance, a MITE insertion in the *ZmNAC111* promoter has been associated with natural variation in maize drought tolerance through the repression of this transcription factor gene via RNA-directed DNA methylation and H3K9 dimethylation (Mao et al., 2015). Furthermore, methylation of a MITE insertion in the *MdRFNR1-1* promoter has been positively correlated with its allelic expression in apple in response to drought stress (Niu et al., 2022). In *O. sativa*, a MITE in the promoter of *HTG3* has been found to be significantly associated with heat-induced expression of *HTG3* and heat tolerance, thus regulating the JASMONATE ZIM-DOMAIN genes (Wu et al., 2022). Additionally, MITEs may be inserted into different positions within genes, interrupting their normal transcription. Our results demonstrate the insertion of a total of 185, 200, and 4302 MITEs into UTR, exon, and intron regions of genes, respectively. A previous study reported that a MITE insertion in the intron of the transcription factor gene *WRKY45-1* generates a small interfering RNA responsible for the negative regulatory role of *WRKY45-1* in suppressing the expression of siR815 Target 1 (Zhang et al., 2016). Furthermore, the insertion of a single copy of mPing into an intron of the photoperiod gene *Hd1* was found to downregulate the expression of the host gene (Yano et al., 2000). To gain an overall perspective of the biological processes associated with MITE-related genes, we conducted GO and KEGG enrichment analyses. The majority of MITE-related genes were found to be associated with various biological processes, with the highest relevance observed for ncRNA metabolic processes, organelle organization, protein-containing complex organization, RNA processing, and others. Therefore, we can speculate that MITE insertions represent potential resources upon which natural and artificial selection can act to influence various biological processes.

In the post-transcriptional regulation of gene expression, mature miRNAs can downregulate target transcripts through mRNA cleavage or translational repression mechanisms (Bartel, 2004; Zhang et al., 2019). Recent studies have provided evidence that certain miRNAs can originate from a group of non-autonomous class II TEs known as MITEs (Crescente et al., 2022). In rice, it has been observed that 80% of TE-derived miRNAs are derived from MITEs, while 10% originate from retrotransposons and 9% from other DNA transposons (Li et al., 2011). To identify miRNAs and their targets, we employed a rigorous approach using miRDeep-P2, implementing a new filtering strategy and improving the algorithm. Unlike previous identification strategies based on sequence similarity, our approach adhered to stringent rules for miRNA and target discovery. Notably, our BLAST-based approach identified a total of 385 miRNAs as originating from MITEs, significantly exceeding the 171 miRNAs identified by miRDeep-P2 (**Supplementary Tables S13**, **S17**). This suggests a higher incidence of false positives in analyses relying solely on sequence similarity. In barley, MITE-derived miRNAs accounted for approximately 7.73% of the total miRNA pool, which is comparable to the proportions observed in *Citrus* species (12.9%) (Liu et al., 2019), *Morus notabilis* (15.9%) (Xin et al., 2019), and *T. aestivum* (14.07%) (Crescente et al., 2022). Considering the high copy numbers of MITEs in the barley genome and their preferential distribution in gene-rich regions, this regulatory network may have a significant impact on post-transcriptional control of gene expression in barley and related species.

Based on the co-linearity-incorporating MITE-based phylogenetic tree, it is evident that barley and rye share a more recent common ancestry. This finding aligns with the results based on orthologous genes, although some differences in the overall topology among all species still exist (Chen et al., 2020b). Importantly, our results indicate that the distance to the common ancestor with barley is not significantly correlated with the proportion of co-linearity-incorporating MITEs. Furthermore, based on the pan-genomic data of barley, the proportion of conserved MITEs with co-linearity is 24.91%, which is substantially lower than the gene proportion of 73.20% (Berthelier et al., 2018). Considering that MITEs possess complete terminal ends that can be mobilized by autonomous molecular mechanisms, their conservation is lower compared to genes. Additionally, MITE insertions in the genome predominantly occur in intergenic regions (Berthelier et al., 2018).

Plant domestication involves the transformation of wild plant species into domesticated crops through artificial selection to induce phenotypic changes (De Leon et al., 2019). This process specifically targets a collection of pivotal agronomic traits collectively known as the “domestication syndrome” (Olsen and Wendel, 2013). In barley, these phenotypic changes encompass grain shattering (Pourkheirandish et al., 2015), caryopsis morphotype (Taketa et al., 2008), and spike morphology, including the fertility of the lateral spikelet in six-row cultivars (Komatsuda et al., 2007; Bull et al., 2017). In our study, we identified a series of MITE insertions/deletions associated with domestication. Specifically, we observed insertions in the promoter region of the transcription factors *HORVU.MOREX.r3.4HG0406410* (*AP2*), *HORVU.MOREX.r3.2HG0204320* (*ARF*), and *HORVU.MOREX.r3.5HG0486320* (*C2H2*). Transcription factor families have been recognized for their significant roles in plant growth, development, and responses to environmental stress (Strader et al., 2022). These MITE insertions associated with domestication provide valuable insights into understanding the artificial domestication of barley, identifying genes with potential applications, and facilitating breeding efforts. However, it is important to emphasize that these results are primarily based on bioinformatics analysis, and experimental validation is essential. Our future work will focus on experimental validation to further support these findings.

## Data Availability

The original contributions presented in the study are included in the article/**Supplementary Material**. Further inquiries can be directed to the corresponding authors.

## Supplementary material

Supplementary Figure 1 Secondary structure of MITEs (Selected examples). **(A)** Secondary structure of *Tc1/Mariner-like* family MITEs. **(B)** Secondary structure of *PIF/Harbinger-like* family MITEs. **(C)** Secondary structure of *hAT-like* family MITEs. **(D)** Secondary structure of *Mutator-like* family MITEs.

Supplementary Figure 2 Comparison of density distribution between genes and MITEs in different barley accessions. **(A–F)** represent the MITE density distribution of the barley genomes Barke, Igri, HOR10350, HOR13942, B1K-04-12 and OUH602, respectively. **(G–L)** correspond to the gene density distribution of in the barley genomes Barke, Igri, HOR10350, HOR13942, B1K-04-12 and OUH602, respectively. The color gradient from blue to red indicates higher densities at the corresponding sites.

Supplementary Figure 3 Spatial distribution of LTR retrotransposons across the barley genome. **(A)** Classification of LTR retrotransposon superfamilies. **(B)** Frequency of LTR retrotransposon insertions near genes in barley. **(C)** Chromosomal density distribution of LTR retrotransposons, with color gradients from blue to red indicating varying densities.

Supplementary Figure 4 miRNA Expression in Different Samples. **(A)** Density plot showing the distribution of miRNA expression levels across ten different samples. **(B)** Box plots representing the variability in miRNA expression levels among the same set of ten samples.
