# Supplementary figures and images for: Genome-wide characterization and evolution analysis of miniature inverted-repeat transposable elements in Barley (Hordeum vulgare)
Source: Front Plant Sci. 2024 Oct 31;15:1474846. doi: 10.3389/fpls.2024.1474846 (PMC11560428; doi:10.3389/fpls.2024.1474846)

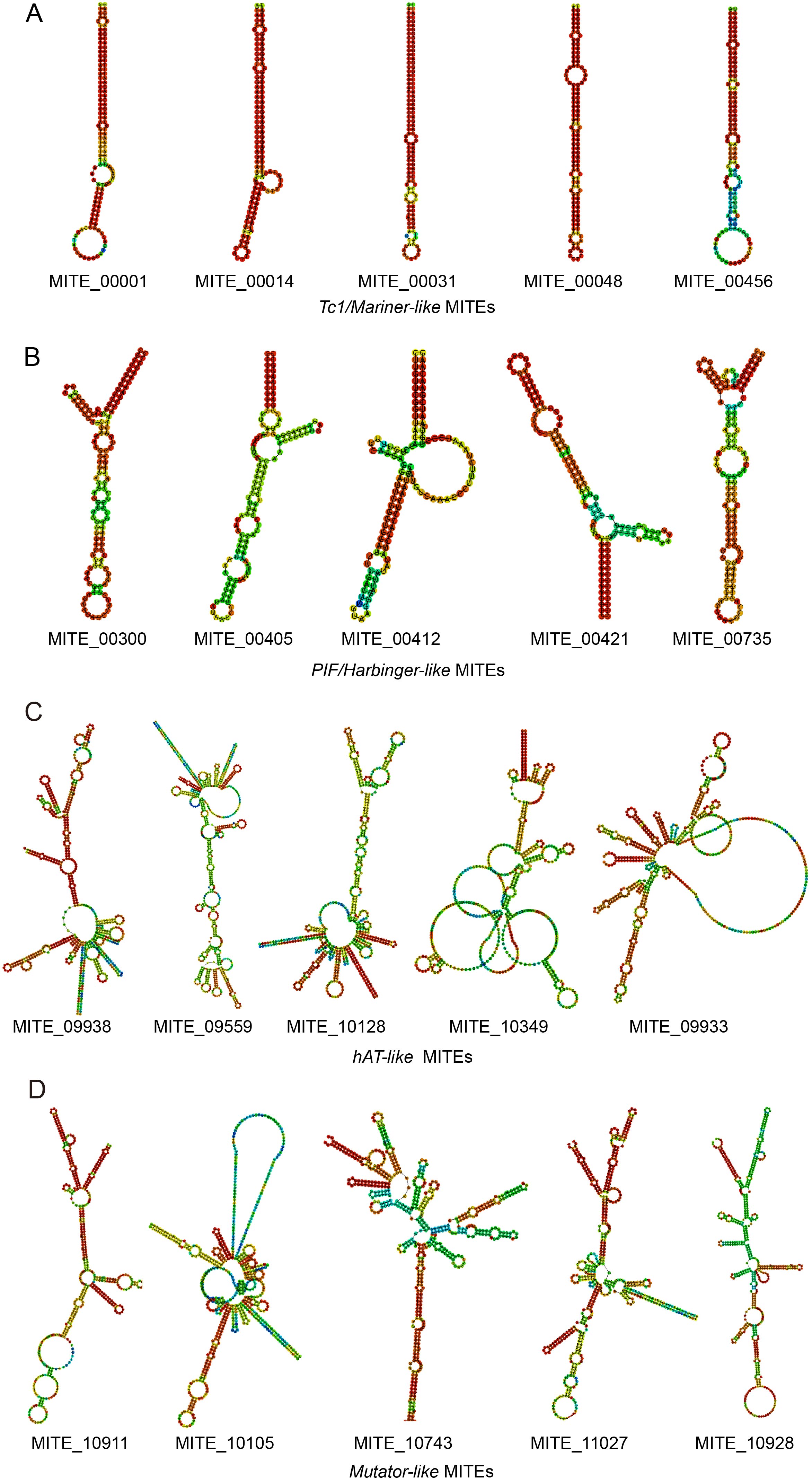

Supplement: Supplementary Figure 1 — Secondary structure of MITEs (Selected examples). (A) Secondary structure of Tc1/Mariner-like family MITEs. (B) Secondary structure of PIF/Harbinger-like family MITEs. (C) Secondary structure of hAT-like family MITEs. (D) Secondary structure of Mutator-like family MITEs. [file Image1.jpeg]

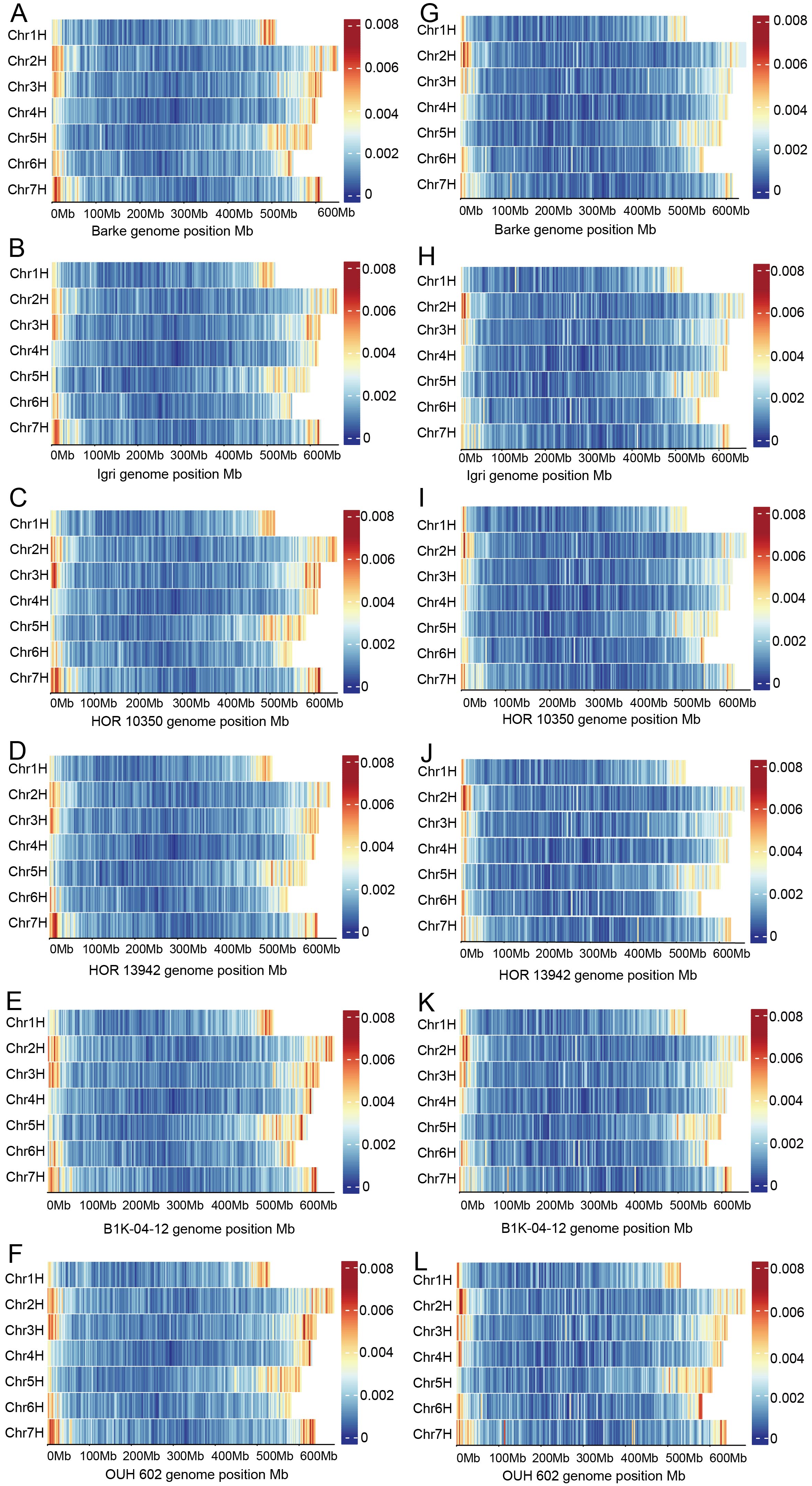

Supplement: Supplementary Figure 2 — Comparison of density distribution between genes and MITEs in different barley accessions. (A–F) represent the MITE density distribution of the barley genomes Barke, Igri, HOR10350, HOR13942, B1K-04-12 and OUH602, respectively. (G–L) correspond to the gene density distribution of in the barley genomes Barke, Igri, HOR10350, HOR13942, B1K-04-12 and OUH602, respectively. The color gradient from blue to red indicates higher densities at the corresponding sites. [file Image2.jpeg]

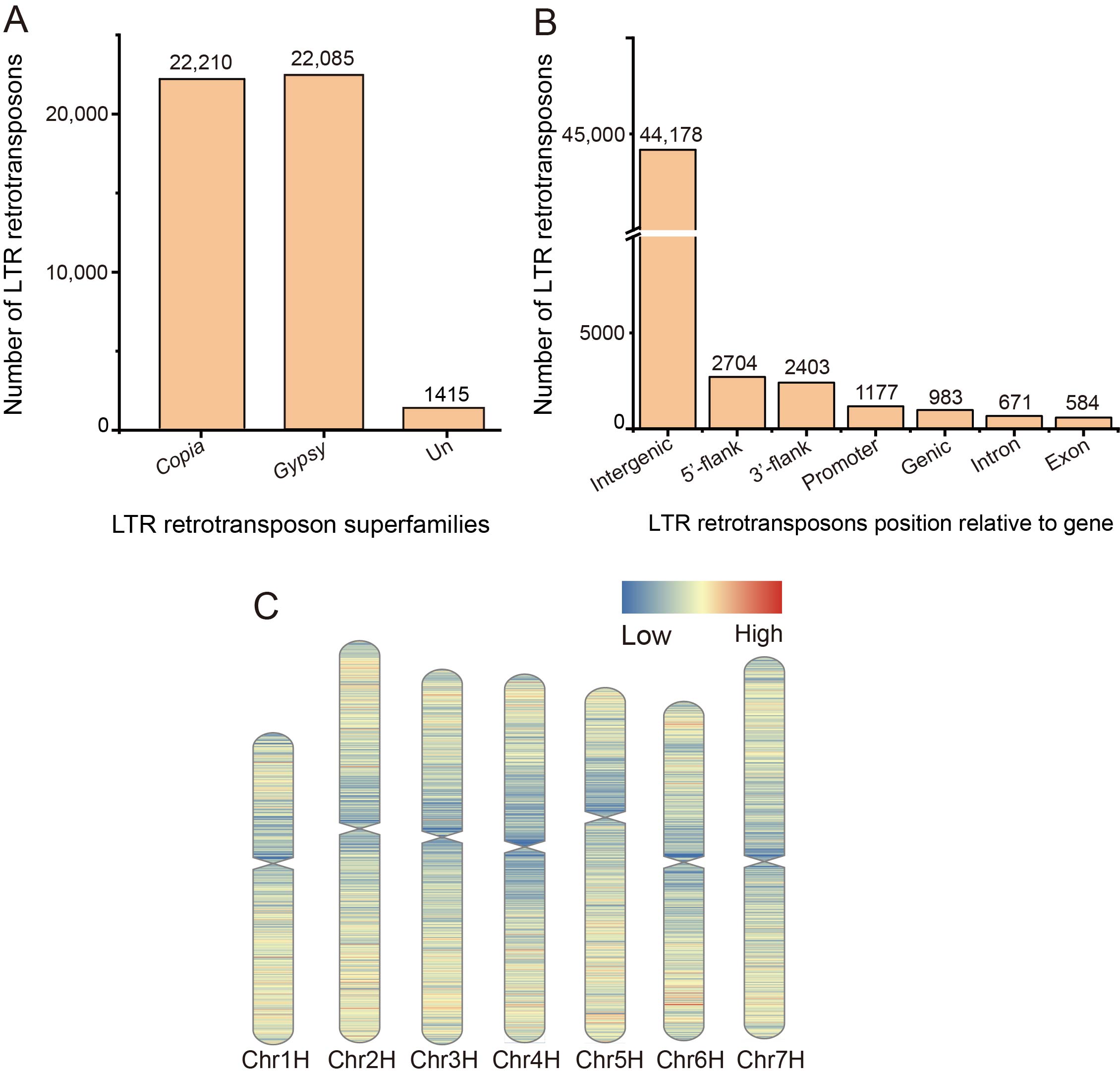

Supplement: Supplementary Figure 3 — Spatial distribution of LTR retrotransposons across the barley genome. (A) Classification of LTR retrotransposon superfamilies. (B) Frequency of LTR retrotransposon insertions near genes in barley. (C) Chromosomal density distribution of LTR retrotransposons, with color gradients from blue to red indicating varying densities. [file Image3.jpeg]

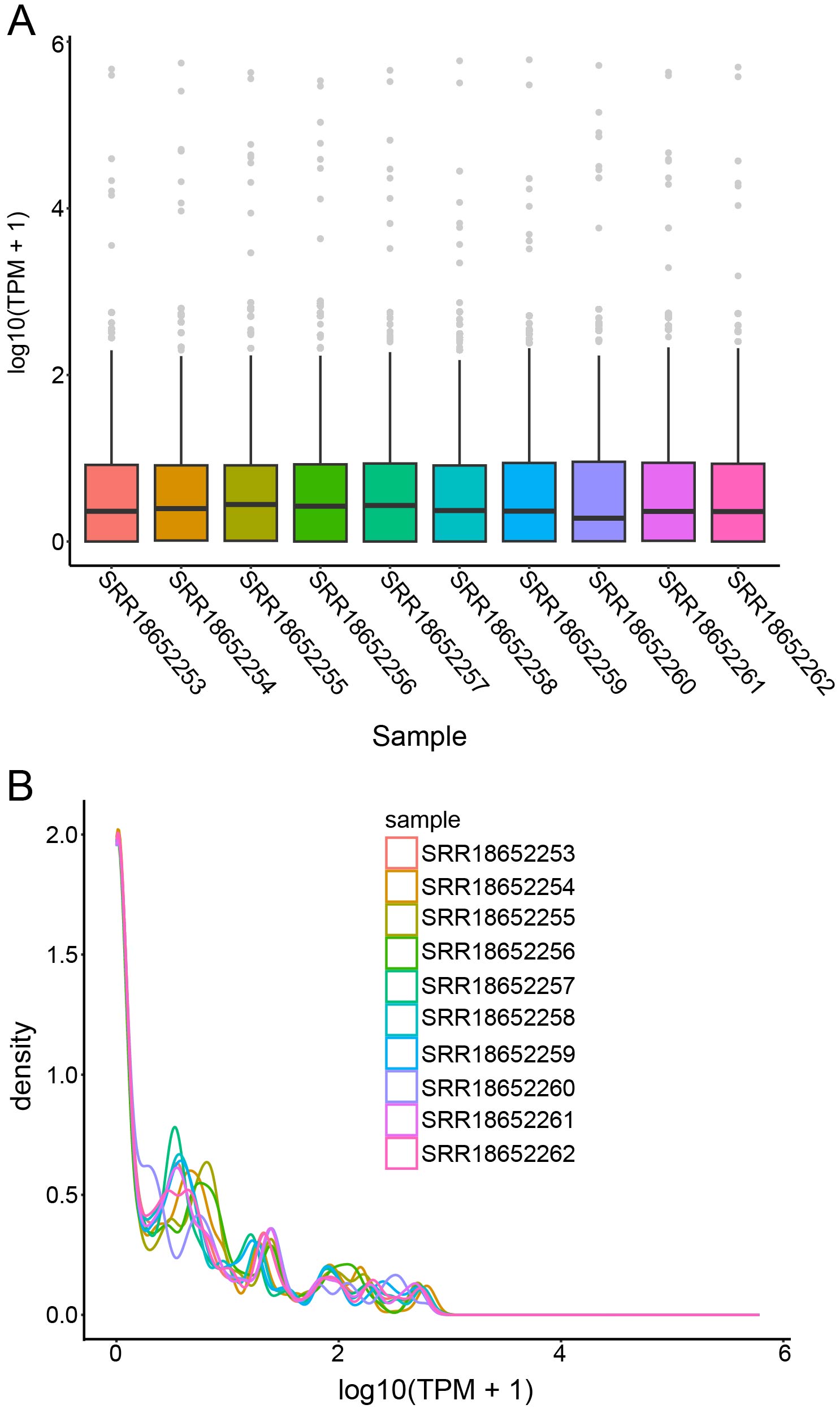

Supplement: Supplementary Figure 4 — miRNA Expression in Different Samples. (A) Density plot showing the distribution of miRNA expression levels across ten different samples. (B) Box plots representing the variability in miRNA expression levels among the same set of ten samples. [file Image4.jpeg]
